# Protein Biochemistry and Molecular Modeling of the Intra-Melanosomal Domain of Human Recombinant Tyrp2 Protein and OCA8-Related Mutant Variants

**DOI:** 10.3390/ijms23031305

**Published:** 2022-01-24

**Authors:** Monika B. Dolinska, Taariq Woods, Isabella Osuna, Yuri V. Sergeev

**Affiliations:** National Eye Institute, National Institutes of Health, Bethesda, MD 20892, USA; dolinskam@nei.nih.gov (M.B.D.); taariq.woods@nih.gov (T.W.); isabella.osuna@nih.gov (I.O.)

**Keywords:** tyrosinase-related protein 2, intra-melanosomal domain, protein expression/purification, protein stability, effect of reducer, Cys-rich domain, OCA8-related mutant variants

## Abstract

Tyrosinase-related protein 2 (Tyrp2) is involved in the melanogenesis pathway, catalyzing the tautomerization of dopachrome to 5,6-dihydroxyindole-2-carboxylic acid (DHICA). Recently, a new type of albinism was discovered with disease-causing mutations in the *TYRP2* gene. Here, for the first time, we characterized the intra-melanosomal protein domain of Tyrp2 (residues 1-474) and missense variants C40S and C61W, which mimic the alterations found in genetic studies. Recombinant proteins were produced in the *Trichoplusia Ni (Ti. Ni)* larvae, purified by a combination of immobilized metal affinity (IMAC) and gel-filtration (GF) chromatography, and biochemically characterized. The mutants showed the protein expression in the lysates such as the wild type; however, undetectable protein yield after two steps of purification exhibited their misfolding and instability. In addition, the misfolding effect of the mutations was confirmed computationally using homology modeling and molecular docking. Together, experiments in vitro and computer simulations indicated the critical role of the Cys-rich domain in the Tyrp2 protein stability. The results are consistent with molecular modeling, global computational mutagenesis, and clinical data, proving the significance of genetic alterations in cysteine residues, which could cause oculocutaneous albinism type 8.

## 1. Introduction

Oculocutaneous albinism (OCA) is a heterogeneous group of rare inherited disorders affecting the biosynthesis of melanin. For many years, seven subtypes of OCA, caused by a mutation in different genes, were known, namely, OCA1 (*TYR*), OCA2 (*OCA2/former* *P* gene), OCA3 (*TYRP1*), OCA4 (*SLC45A2*), OCA5 (*OCA5*), OCA6 (*SLC24A5*), and OCA7 (C10ORF11) (OMIM^®^, An Online Catalog of Human Genes and Genetic Disorders; http://www.omim.org; accessed on 20 January 2022). Recently, the new mutations in the *TYRP2* gene (also known as the *DCT* gene), involved in albinism, were identified in the genetic study [1]. Pennamen et al. showed that mutations in the *DCT* gene, C40S, and C61W can cause a new type of oculocutaneous albinism, which they proposed to name OCA8. Moreover, Volk et al. identified the new mutation in the *TYRP2* gene, G59V [2]. From their research, OCA8 appears as a type of OCA group with a mild to moderate range on the phenotypical spectrum. Loss of function of Tyrp2 protein was also investigated in mice and zebrafish [1]. When introduced in mice, C40S and C61W mutations result in hypopigmentation of the coat. In early zebrafish embryos, Tyrp2 insufficiency impairs melanin metabolism in both melanophores and retinal pigment epithelium (RPE) cells with possible consequences on eye development and/or function.

Tyrp2, also called dopachrome tautomerase (DCT) (EC 5.3.3.12), together with tyrosinase (Tyr) and tyrosinase-related protein 1 (Tyrp1), is one of the key melanocyte-specific enzymes in the mammalian melanogenesis pathway. Its catalytic activity was first reported by Pawelek et al. in 1980 [3]. Tyrp2 catalyzes tautomerization of the pigmented intermediate dopachrome to DHICA, which regulates the proportion of carboxylated subunits in the melanin biopolymer [4]. Without Tyrp2 present, dopachrome can generate DHICA in the presence of divalent metal cations or spontaneously lose its carboxyl group to form the 5,6-dihydroxyindole (DHI) [5,6,7,8]. This could result in the induction of the brown, poorly soluble, intermediate MW DHICA-delivered melanin and black, insoluble, high MW DHI melanin [9,10]. However, DHI forms reactive oxygen species (ROS). Tyrp2 preserves the carboxylic acid group of dopachrome, which protects the cells from the toxic effect of the ROS formation and plays a critical role in lowering the oxidative stress resulting from melanogenesis [11,12]. On the other hand, the absence of Tyrp2 is not as critical for melanin formation as the absence of Tyr. It does not lead to a loss of pigmentation, but rather to a color change.

Tyrp2 is a transmembrane glycosylated protein containing two zinc ions, ZnA and ZnB, coordinated by six histidine residues that resided in a cavity of the active site. Like Tyr and Tyrp1, before arriving in the melanosomes, it undergoes N-glycosylation and then moves from the endoplasmic reticulum (ER) to the Golgi; however, when immature, stays in the ER [13]. In mutant protein, the G59V variant is exclusively colocalized subcellular with the ER marker, calnexin, and did not reach the melanosomes as the wild-type protein, indicating an incomplete protein maturation [2].

Tyrp2 shares approximately 48.76% sequence identity with Tyrp1 and 39.85% with Tyr (https://blast.ncbi.nlm.nih.gov/Blast.cgi; accessed on 20 January 2022) (Figure S1). All three tyrosinases have many common features including localization to the melanosome, the C-terminal transmembrane helices, tyrosinase, and Cys-rich domains, as well as similar N-glycosylation sites. All these proteins were previously purified as active enzymes and characterized [12,14]; however, the only crystal structure of Tyrp1 is currently available [15]. It is believed that in the melanosome membrane, all three proteins might form a heterotrimeric Tyr/Tyrp1/Tyrp2 complex, which can play a role in the stabilization of Tyr and regulation of its enzymatic activity [16]. Our recent results in vitro showed no evidence of stable hetero-oligomer formation between Tyr and Tyrp1 intra-melanosomal domains [17]. Therefore, the hetero-trimeric complex might be formed due to intra-membrane interactions of transmembrane helices of Tyr, Tyrp1, and Tyrp2 proteins [16]. We also showed in vitro the links between OCA1-related mutations, Tyr conformational stability, and enzymatic activity [18]. Using computational methods, we identified mechanisms behind mutant variant instability and differences behind protein–ligand interactions in Tyrp1 and OCA3 mutations [19].

Here, for the first time, we characterized the intra-melanosomal protein domain of Tyrp2 (residues 1-474) and missense variants C40S and C61W, which mimic the alterations found in genetic studies [1]. Recombinant proteins were produced in the *Ti. Ni* larvae, purified by a combination of affinity and size-exclusion chromatography and characterized by protein biochemistry methods. Moreover, previously we demonstrated in vitro and with molecular modeling that some mutations in the Cys-rich domain are critical for tyrosinase stability and are related to OCA3 disease [19]. Using in vitro and computational methods, we confirmed the importance of the Cys-rich domain for the Tyrp2 protein stability and a role of recently discovered OCA8-related mutations: C40S, C61W, and G59V. This study proposes that the Tyrp2 genetic mutations impact protein folding and stability.

## 2. Results

### 2.1. Protein Purification

**Figure 1:**
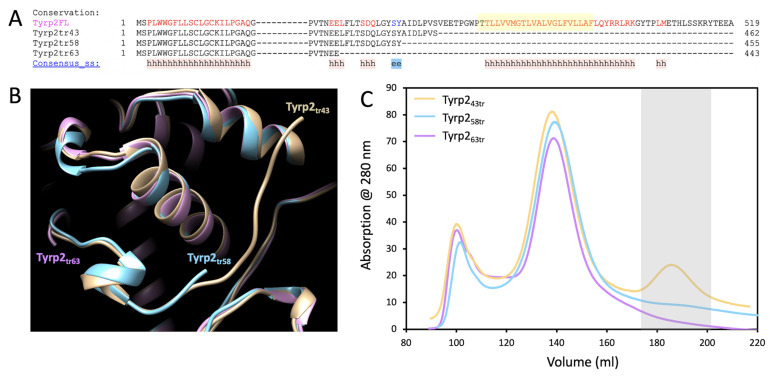
Stability of the human recombinant Tyrp2 proteins with different truncation sites. (**A**) The PROMALS3D sequence alignment (http://prodata.swmed.edu/promals3d/promals3d.php; accessed on 20 January 2022) of human Tyrp2 full length (Tyrp2FL, P40126 UniProtKB) and the Tyrp2 sequences with three amino acid truncation lengths: 43 (Tyrp2tr43), 58 (Tyrp2tr58), and 63 (Tyrp2tr63)_._ Representative Tyrp2FL sequence (magenta) is colored according to predicted secondary structures (red: alpha-helix, blue: beta-strand). Symbols for consensus predicted secondary structure (Consensus_ss) is h for alpha-helix and e for beta-strand. Highlighted with yellow is the transmembrane domain of Tyrp2FL. (**B**) The superimposed structures of Tyrp2_tr43_ (tan ribbon), Tyrp2_tr58_ (blue ribbon), and Tyrp2_tr63_ (pink ribbon). (**C**) The chromatograms of Tyrp2_tr43_ (yellow), Tyrp2_tr58_ (blue), and Tyrp2_tr63_ (pink) after two steps of purification (IMAC and GF) using the His-Trap Crude 5 mL and HiPrep 26/60 Sephacryl S-300 columns on the ÄKTAxpress chromatography system. The picks of protein of interest are in the grey box.

Here, three recombinant proteins with the successive three C-terminal truncations of the human Tyrp2, Tyrp2_43tr_, Tyrp2_58tr_, and Tyrp2_63tr_ were expressed in *Ti. Ni* larvae as previously described [14,20]. The multiple protein sequence alignment of protein sequences and structural superposition of truncated protein structures is shown in Figure 1A,B. At the second protein purification step, when proteins were eluted from the Sephacryl S-300 HR column, three peaks were observed. The first two peaks corresponded to the void volume of the column, which contained a wide range of proteins with an MW of 20–300 kDa. These two peaks were nearly identical for chromatography profiles of Tyrp2_43tr_, Tyrp2_58tr_, and Tyrp2_63tr_. The third peak was present only in the Tyrp2_43tr_ chromatography profile and was absent in the profiles of the two other proteins, Tyrp2_58tr_ and Tyrp2_63tr_ (Figure 1C).

**Figure 2:**
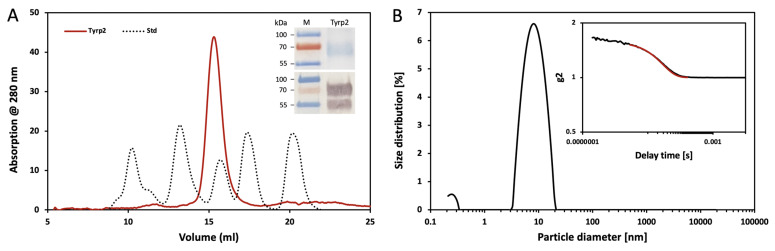
Monomeric stage of Tyrp2 protein. (**A**) Chromatography profile of Tyrp2 for fractions eluted from the Superdex 200 Increase GL 10/300 column (red). The dotted line corresponds to the Bio-Rad GF standards. From the left: thyroglobulin (670 kDa), γ-globulin (158 kDa), ovalbumin (44 kDa), myoglobin (17 kDa), and vitamin B12 (1.3 kDa). Insert shows the SDS-PAGE (top panel) and the Western blot (bottom panel) of Tyrp2. The left line displays the protein ladder marker (M) at 55, 70, and 100 kDa. (**B**) Dynamic light scattering of Tyrp2 protein. Particle diameter distribution by intensity for Tyrp2 at 1 mg/mL measured at 25 °C. Insert shows the correlation function, where g2 is the correlation function intercept. The measured autocorrelation function is black, and the cumulant fit function is red.

Therefore, for the Tyrp2 protein characterization, the Tyrp2_43tr_ (hereafter, ‘Tyrp2′) variant was used. After purification by IMAC and GF chromatography, Tyrp2 was eluted from the Superdex 200 Increase 10/300 GL column as a monomer, with an apparent molecular weight of 54.3 ± 0.9 (Figure 2A, Table 1) and migrated from SDS-PAGE gel as the broad, heterogeneous bands between 55 and 70 kDa, which reacted strongly (as a double band) with anti-Tyrp2 antibody (B7, Santa Cruz Biotechnology) (Figure 2A insert). This could be attributed to the N-linked glycosylation or may indicate the protein’s isoforms. To further prove the monomeric state of Tyrp2, we performed dynamic light scattering (DLS) (Figure 2B). The hydrodynamic diameter of Tyrp2 suspended particles was calculated, showing a monomodal size distribution with an average particle size of 9.23 ± 0.39 nm and a mean hydrodynamic diameter of 7.76 ± 0.18 nm (Figure 2B, Table 1).

The purified human recombinant Tyrp2 protein was tested using inductively coupled plasma-mass spectrometry (ICP-MS) for the presence of Cu^2+^ and Zn^2+^ ions bound to the active site (Table 1). According to the data, a sample of Tyrp2 contained both Cu^2+^ and Zn^2+^ ions, which were determined to have concentrations above the detection limits of 0.001 and 0.01, respectively. However, the level of Zn^2+^ ions was five times higher than the level of Cu^2+^. In contrast, for Tyrp1, the level of Zn^2+^ ions was more than 50 times higher than the level of Cu^2+^, and only Cu^2+^ ions were detected in Tyr.

### 2.2. Enzymatic Activity

**Figure 3:**
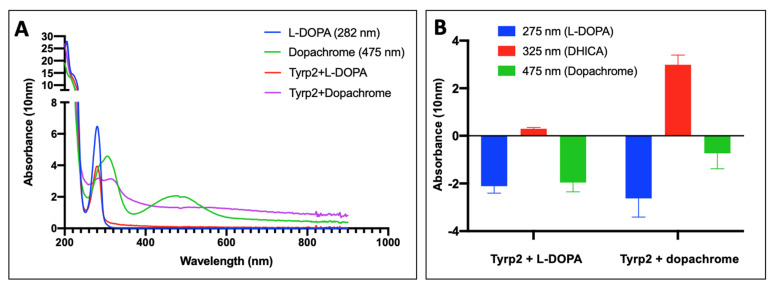
Enzymatic activity of Tyrp2. (**A**) The spectrum that represents the recorded wavelengths from 200–900 nm for L-DOPA (blue), dopachrome (green), Tyrp2 in the presence of L-DOPA (red), and Tyrp2 in the presence of dopachrome (purple) over three separate experiments. (**B**) A graph that represents the 30 min reaction of Tyrp2 in the presence of L-DOPA (bars 1–3 from left) or dopachrome (bars 4–6 from left) measured at 275 nm (L-DOPA, blue bars), 325 nm (DHICA, red bars), and 475 nm (dopachrome, green bars) over three separate experiments with the baseline subtracted. The error bars represent the standard error of the mean (SEM).

Tyrp2 catalyzes the tautomerization of pigmented intermediate dopachrome to carboxylate derivative of DHICA. Here, Tyrp2 was incubated with L-DOPA or dopachrome for 1 h at 37 °C, and the absorption spectrum for the wavelength in the range of 200–900 nm was recorded (Figure 3A). Dopachrome was produced in the reaction of L-DOPA with Tyr immobilized to the magnetic beads [21]. In the presence of L-DOPA, the spectrum was on the baseline level; however, in the presence of dopachrome, two additional peaks appeared at 325 and 560 nm, which corresponded to DHICA and indole-5,6-quinone-2-carboxylic acid (IQCA), respectively. When the baseline was subtracted, the significant level of DHICA only was detected, while Tyrp2 was incubated in the presence of dopachrome (Figure 3B).

### 2.3. Tyrp2 Instability under Reducing Conditions

**Figure 4:**
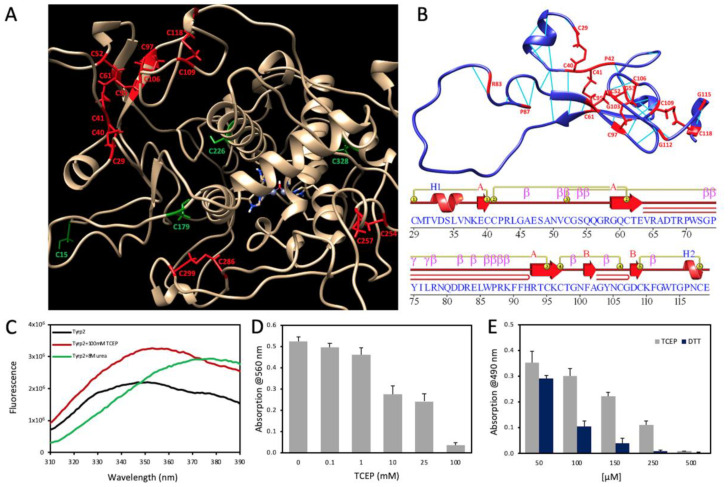
Influence of reducing agents on Tyrp2 stability and dopachrome tautomerase activity. (**A**) Cys residues of Tyrp2 mapped to the human 100 ns Tyrp2 structure (gold ribbon) modeled as described in the Materials and Method section. Cysteines involved in disulfide bridges are red, and the intact Cys residues are green. (**B**) Homology model (top panel) and topology (bottom panel) of the Cys-rich domain of Tyrp2. The homology model is colored accordingly to the residue foldabilities. Residues with foldability 19 are critical for domain stability (red). In the bottom panel, elements of the secondary structure are in red. Disulfide bridges are yellow lines. (**C**) Intrinsic tryptophan fluorescence spectra of the native Tyrp2 are shown by the black line. Tyrp2 spectra are shown after the incubation for 1 h in 100 mM TCEP (red line) and 8 M urea (green line). Emission of 5 μM protein samples excited at 280 nm registered between 300 and 400 nm. (**D**) Dopachrome tautomerase activity of Tyrp2 decreases with the increase of the reducing agent (TCEP). Activity measured spectrophotometrically (absorption at 560 nm) in 50 mM Tris buffer, pH 7.4, after 1 h of incubation at 37 °C in the presence of TCEP in the range of 0–100 mM. Tyrp2 concentration was 0.25 mg/mL. (**E**) Inhibitory effect of TCEP (grey bars) and DTT (dark blue bars) reducing agents on diphenol oxidase activity of Tyr. Activities were measured spectrophotometrically (absorption at 490 nm) in 50 mM sodium phosphate buffer, pH 7.5, after 30 min of incubation with reducers (0–500 mM) at 37 °C. Tyr concentration was 0.5 mg/mL.

In Tyrp2, cysteines formed seven disulfide bridges localized in Cys-rich (C29/40, C41/61, C52/95, C97/106, C109/118) and tyrosinase (C254/257 and C286/299) domains (Figure 4A). All cysteines in the Cys-rich domain were critical for Tyrp2 protein stability, as shown in Figure 4B in red. To understand the role of the Cys-rich domain and disulfide bridges, we used tris(2-carboxyethyl)phosphine (TCEP) to monitor the conformational changes of Tyrp2 and its activity under different reducing conditions. In this experiment, the intrinsic tryptophan fluorescence of Tyrp2 was measured under the native, reduced (in the presence of TCEP), and chemical unfolding (in the presence of urea) conditions (Figure 4). Intact Tyrp2 showed emission maximum at 347 nm when tryptophan residues were in a polar environment inside the protein structure (Figure 4C). When Tyrp2 was incubated with 8 M urea and when the buried and quenched tryptophan residues became solvent-exposed, we observed a redshift to 377 nm with an increase in intensity. A smaller redshift to 353 nm was observed for Tyrp2 under reducing conditions. Tyrp2 dopachrome tautomerase activity was inhibited by TCEP (Figure 4D), similarly to the TCEP and DTT inhibition of Tyr diphenol oxidase activity (Figure 4E).

### 2.4. Disease-Related Mutant Variants

**Figure 5:**
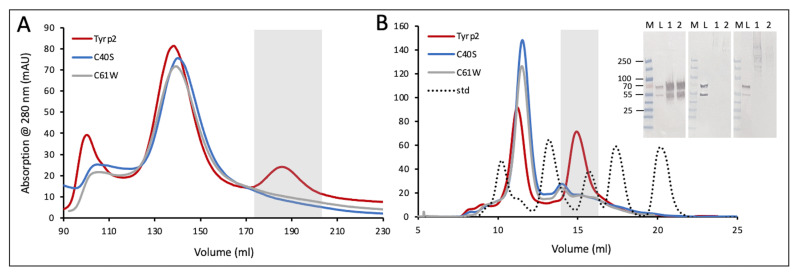
The stability of the human recombinant Tyrp2 and OCA8-related mutant variants. Chromatography profiles of Tyrp2 (red) and mutant variants (C40S, blue, and C61W, grey) eluted from the Sephacryl S-300 HR column followed after IMAC (**A**) and Superdex 200 Increase GL 10/300 column (**B**). The picks of protein of interest are in grey boxes. The black dotted line corresponds to the Bio-Rad GF standards. Insert shows the Western blot of Tyrp2, and OCA8-related mutants using anti-Tyrp2 B7 antibody. Inserts from the left: Tyrp2, C40S, and C61W. Lanes in each insert: M, protein ladder; L, lysate; 1, Tyrp2; 2, C40S, and 3, C61W.

To investigate the effect of genetic mutations on the human Tyrp2 structure and stability, we expressed and purified two OCA8-related mutants (C40S and C61W). The missense mutations were created on the basis of the Tyrp2_43tr_ construct. In contrast to the Tyrp2 wild type, mutants showed no peak when eluted from GF columns (Figure 5) and indicated no bands at the proper position on the Western blots (Figure 5B inset). This indicates that both mutant variants showed no soluble protein (Table 2). Finally, C40S and C61W mutant variants appeared to be misfolded and aggregated.

**Figure 6:**
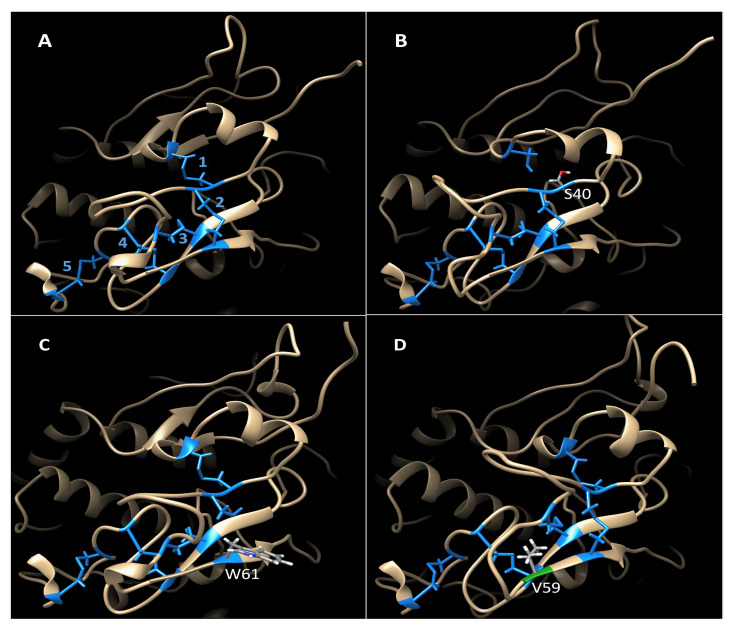
Cys-rich domains of Tyrp2 and mutant variants. (**A**) Tyrp2 structure at 50 ns with numbered disulfide bridges. (**B**) C40S mutant variant at 50 ns. The introduction of serine at position 40 prevented the formation of disulfide bridge 1. (**C**) C61W mutant variant at 50 ns. The introduction of tryptophan at position 61 prevented the formation of disulfide bridge 2. (**D**) G59V mutant variant at 50ns. Mutation to valine at position 59 (green) did not alter the formation of disulfide bridges in the structure.

Disease-related mutant variants were analyzed computationally to understand their protein stability. Each Tyrp2 residue had a foldability parameter or an aggregated sum of the propensities of the unfolding parameters of that residue’s mutation. The foldability parameter highlights the most critical residues involved in the thermodynamic stability of the protein. In Figure S2, the foldability pattern is shown for the Tyrp2 homology model. Residues critical for protein stability are shown in red and localized in the central part of the protein molecule. Alterations at these positions could potentially destabilize a protein fold. As shown in Table 2, both C40 and C61 were expected to contribute the most to protein stability, with foldability values of 19. These values were consistent with the unfolding parameters of 1 and 0.99 for the C40S and C61W mutations, respectively, indicating a complete misfolding of the mutant. These results matched the pathogenicity predictions from ClinVar and the results of the mutants’ expression/purification (https://www.ncbi.nlm.nih.gov/clinvar; accessed on 20 January 2022) (Table 2). The third mutation, G59V, was also expected to be a misfolding-causing mutation. Unfortunately, we currently do not have clinical and biochemical data about the effect of this mutation. The mutation G59V resulted in incomplete protein maturation and targeting in vitro compatible with a partial or total loss of function [2]. The superpositions of wild-type Tyrp2 model and models of mutant variants are shown in Figure 6A–D. In addition, all cysteine residues, which were forming disulfide bridges, had high foldability parameters (Table 2) and were conserved from an evolutionary perspective (Figure S9). The importance of cysteine mutations agreed with phenotypic changes in homozygous mice with C40S and C61W missense mutations in the Tyrp2 gene (equivalent to those found in patients with OCA8), exhibiting dark gray hair and significantly less pigmentation of the RPE [1].

Protein residue–residue distance maps were created in search of structural changes, which occurred during molecular dynamic simulations (Figures S3–S5). The changes of the electrostatic potential at the protein surface of Tyrp2 and mutant variants are shown in Figure S8.

**Figure 7:**
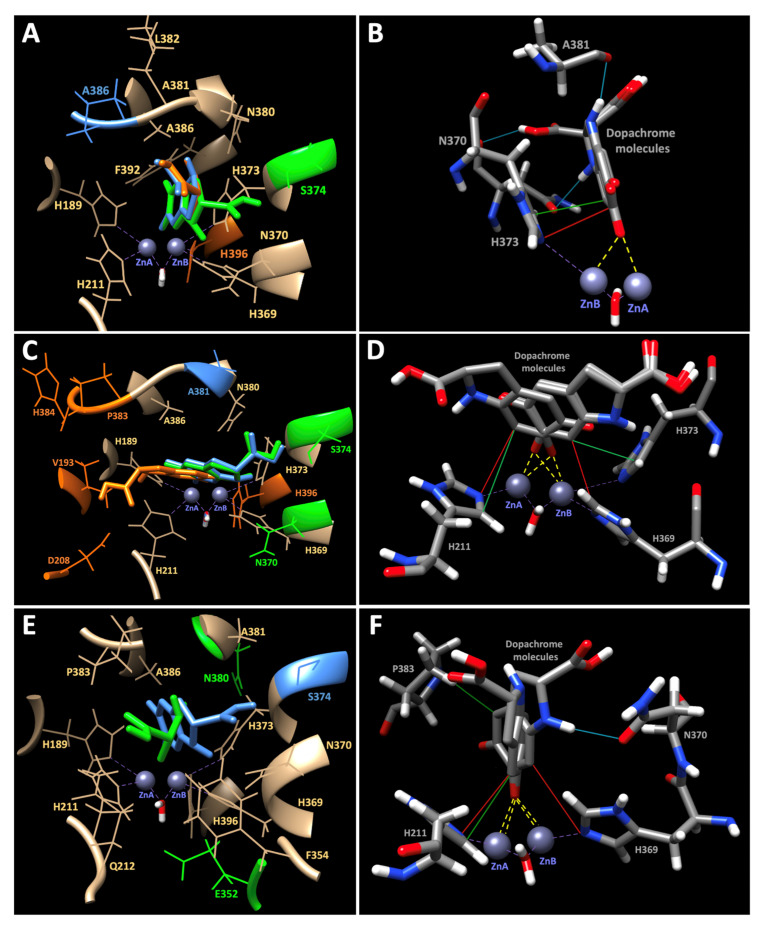
Computational docking of dopachrome molecule to human Tyrp2 and OCA8-related mutants. Dopachrome docked in different poses (show as green, orange, and blue molecules) with Tyrp2 (**A**), C61W (**C**), and G59V (**E**) visualized using UCSF Chimera 1.15.0. All contacting receptor residues, which are common for all poses, are colored tan. Contacting residues specific for the pose are colored with the same color as the molecule. (**B**,**D**,**F**) show the dopachrome interactions: hydrogen bonds (blue lines), hydrophobic (green lines), and *π–π* interactions (red lines). The distances between the docked molecule and Zn atoms, shown as purple spheres, are colored yellow. The dopachrome molecules and contacting residues are colored by elements: carbon with grey, nitrogen with blue, oxygen with red, and hydrogen with white.

Furthermore, we looked for the most stable conformations of the mutant binding sites and docked Tyrp2 ligand to understand a possible mechanism of Tyrp2 catalytic activity (Figure 7). Dopachrome, the substrate of Tyrp2 enzyme, was docked to Tyrp2, C40S, C61W, and G59V using VINA software implemented in YASARA 21.8.2. Docking results were considered valid only if the oxygens on the aromatic ring of dopachrome were oriented towards the active site of a protein and were at the distance <4Å from zinc molecules (Figure 7). Three properly oriented poses were found for Tyrp2 and C61W, and two for G59V. For the C40S mutant variant, no docking to the active site was observed. For Tyrp2/dopachrome, the binding energy was found to be 6.59 ± 0.40 kcal/mol, and the dissociation constant 16.66 ± 8.50 µM (Table S1). The ligand–enzyme complex was stabilized by two hydrogen bonds with N370 (O/N370-H7/dopachrome and OD1/N370-H4/dopachrome) or one with A381 (O/A381-H4/dopachrome), hydrophobic interactions (CD2/H373-C6/dopachrome and CD2/H373-C5/dopachrome), and *π–π* interactions (NE2/H373-C8/dopachrome and CD2/H373-C8/dopachrome). The distance from the oxygen atom on the aromatic ring of dopachrome to each of the Tyrp2 zinc atoms changed at ≈2.4 Å. In addition, five of the six histidines, which stabilized the active site, were among the contacting receptor residues. For C61W/dopachrome the binding energy was found to be 6.17 ± 0.23 kcal/mol, and the dissociation constant 31.88 ± 13.54 µM (Table S2). The ligand–enzyme complex was not stabilized by any hydrogen bonds but only by the hydrophobic interaction (CD2/H373-C6/dopachrome, CD2/H211-C6/dopachrome, and CD2/H373-C6/dopachrome) and *π*–*π* interactions (NE2/H369-H6/dopachrome and NE2/H211-C6/dopachrome). The distance from the oxygen atom on the aromatic ring of dopachrome to zinc atoms fluctuated between 2.3 and 2.5 Å. The involvement of the zinc-surrounding histidine residues was varied with the poses. For G59V/dopachrome, the binding energy was found to be 6.32 ± 0.14 kcal/mol, and the dissociation constant 23.65 ± 5.70 µM (Table S3). The distance from the oxygen atom on the aromatic ring of dopachrome to both zinc atoms was ≈2.3 Å, and the ligand–enzyme complex was stabilized by a hydrogen bond with N370 (OD1/N370-H4/dopachrome), by hydrophobic interactions with H211 or P383 (CD2/H211-C5/dopachrome and HG/P383-H5/dopachrome), and *π*–*π* interactions with H311 or H369 (NE2/H211-C5/dopachrome and NE2/H369-C6/dopachrome).

## 3. Discussion

Tyrp2 is involved in the melanogenesis pathway, catalyzing the tautomerization of dopachrome to DHICA. Here, we expressed and produced in *Ti. Ni* larvae, then purified, three different truncated variants of the intra-melanosome Tyrp2. However, only the variant, which was truncated similarly to the Tyr intra-melanosomal domain (just several amino acids at the C-terminus before the transmembrane domain) was stable and properly folded (Figure 1 and Figure S1). The purified Tyrp2 was a monomeric ≈54 kDa glycoprotein (Figure 2, Table 1), similarly to our previously characterized intra-melanosome domains of Tyr and Tyrp1 [14,22]. The metal-binding analysis using ICP-MS showed that Tyrp2 contains zinc atoms in its active site instead of coppers making Tyrp2 the catalyst in the tautomerization versus the copper-mediated oxidation catalyzed by Tyr [23]. This was shown using L-DOPA and dopachrome, the substrates of Tyr and Tyrp2, respectively. When incubated with dopachrome, which was produced in the lab using Tyr immobilized to the magnetic beads [21], two additional peaks appeared at 325 and 560 nm, which corresponded to the DHICA and IQCA, respectively; however, after the subtraction of the baseline, a significant level of DHICA only was detected (Figure 3B).

It has been shown that Tyr catalytic activity is decreased in reducing conditions. Tyrosinases from different sources are inhibited by reducing agents such as DTT or β-ME, which are commonly used to prevent the formation of intramolecular disulfide bonds in proteins [24]. Moreover, we demonstrated that the intra-melanosomal domain of Tyr is strongly inhibited by DTT and β-ME [14]. Like Tyr, Tyrp2 has 18 cysteines. Some of them form disulfide bridges localized in Cys-rich and tyrosinase domains. Therefore, such residues are critical for Tyrp2 protein stability (Figure 4). The conformational changes of Tyrp2 under different reducing conditions we monitored using a reducing agent, TCEP, and intrinsic tryptophan fluorescence. Upon protein chemical unfolding with 8 M urea, when the buried and quenched tryptophan residues became solvent-exposed, we observed a 30 nm redshift, but also a smaller 24 nm red shift was observed for Tyrp2 under reducing conditions. Tyrp2 dopachrome tautomerase activity was inhibited by TCEP, similarly to the TCEP and DTT inhibition of Tyr diphenol oxidase activity (Figure 4). Likewise, this experiment confirmed the role of disulfide bridges as the stabilizers of Tyrp2 protein domains.

The importance of the Cys-rich domain and disulfide bridges for the stability and activity of Tyrp2 was also demonstrated by analyzing its missense variants C40S and C61W, which mimic the alterations found in genetic studies [1]. The recombinant mutants showed the protein expression in the lysates such as the wild-type Tyrp2; however, undetectable protein yield after two steps of purification, exhibiting their misfolding and instability. The misfolding effect of the mutations was confirmed computationally using homology modeling, global mutagenesis, and the pathogenicity predictions from the genetic study (Table 2). Two mutations, C40S and C61W, break the C29-C40 and C41-C61 disulfide bridges, respectively. The Cys-rich subdomain of Tyrp2 is an extremely stable portion of the protein, and a loss of a disulfide bridge may destabilize the protein domain and introduce structural flexibility to this region. The short time (<100 ns) MD simulation did not show the mutant variant instability. However, in the experiment, the mutation could alter the folding process that continued for about 1 ms while C40S or C61W were synthesized in the ER, resulting in a misfolded protein. The variant G59V did not break any disulfide bridge but decreased the conformational flexibility in the loop adjacent to the β-strand. Interestingly, all four transmembrane helices and the zinc–water–zinc complex showed no significant changes caused by genetic mutations (Figures S6 and S7). Residue–residue distance maps of the mutants did not suggest structural fluctuations, which was not evident for Tyrp2. However, since the mutants are expected to be misfolded, the lack of trends towards structural alterations indicates that due to the mutation in the Cys-rich domain, the protein may be unable to properly complete the folding process as it is synthesized in the ER. Taking everything together, experiments in vitro and computer simulations indicated the critical role of the Cys-rich domain in the Tyrp2 protein stability.

Another interesting aspect is the possibility of ‘kinetic trapping’ of mutant Tyrp2 in a non-native protein conformation. Indeed, the mutated variant could be trapped in the non-native protein conformation if the change in native protein leads to a stable protein structure with the lowest free energy (ΔΔG < 0). According to our calculations, the alteration of a cysteine residue to serine or tryptophane (C40S and C61W) suggests an unfolding propensity of 1 (ΔΔG > 2.75 kcal/mol, Table 2). This propensity indicates that the mutant protein is in an unfolded state and degraded and/or aggregated in ER. This conclusion was confirmed by Western blot, demonstrating the absence of soluble fraction and the presence of insoluble protein in the protein supernatant. Therefore, the ‘kinetic trapping’ of the mutant variant in a stable non-native protein conformation seems to not be possible.

Enzymatic activity of the protein is generally associated with its 3D structure, especially in the active site region, and must be conserved to maintain the enzyme-specific function. The missense mutation, even if not directly affecting the active site, can alter the protein stereochemistry around it. The electrostatic potential at the protein surface is a complementary indicator of such alterations. The surface of the Tyrp2 wild type revealed open access to both Zn atoms surrounded by the histidines (Figure S8A). All three mutations—C40S, C61W, and G59V—affecting electrostatic potential distribution in the vicinity of an active site, as shown in Figure S8B–D, demonstrated some changes of the potential at the protein surface. In addition, the electrostatic surface coloring exhibited the increase of positive charge of potential energy around the Tyrp2 active site when protein was mutated. As the exact mechanism of Tyrp2 and OCA8-related mutant’s catalytic activity is not fully understood, we used molecular docking to model the interactions between dopachrome and the proteins at the atomic level (Figure 7, Tables S1–S3). Although the binding energies for Tyrp2, C61W, and G59V were similar, the dissociation constant (Kd) was lower for Tyrp2 than for mutants, suggesting that the dopachrome binds the most readily to the wild-type protein. Moreover, dopachrome docked to Tyrp2 was stabilized by hydrogen bonds, hydrophobic interactions, and *π–π* interactions. These bonds and interactions were all found to have properly oriented poses and were not varied, as in the case of mutants. Furthermore, no docking to the active site was observed for the C40S mutant variant. This result might indicate that the changes in the Cys-rich domain could be critical for the Tyrp2 protein stability.

In our work, we demonstrated that protein stability is compromised in both mutant variants C40S and C61W compared to that of wild-type Tyrp2 and the unstable proteins were processed by protein quality control. The change in protein structure originated by genetic mutation causes OCA8. This association between mutation and disease phenotype is known as the genotype-to-phenotype relationship. Establishing such relationships is an important task in modern medicine. However, the quantitative modeling of the genotype-to-phenotype relationships requires high-quality genetic and clinical data. For example, the molecular modeling suggested an association between the predicted structural alteration and/or damage to protein retinoschisin (RS1) and the severity of XLRS, as measured by the electroretinogram analogous to the RS1-knockout mouse [25,26]. The success of this work was determined by the high quality of clinical data describing retinal function and the electroretinogram a- and b-waves. Unfortunately, OCA is a more complex disease with multiple phenotypic changes. In OCA8, as we mentioned in the Introduction section, the OCA8 phenotypical spectrum is characterized by hair and skin hypopigmentation and ocular features such as nystagmus, foveal hypoplasia, iris transillumination, hypopigmentation of the retina, photophobia, and reduced visual acuity [1]. This wide phenotypical spectrum currently makes it difficult for the further modeling of the genotype-to-phenotype relationships in oculocutaneous albinism.

In summary, for the first time, we expressed and analyzed the intra-melanosomal domain of human recombinant Tyrp2 and OCA8-related mutant variants that have been recently discovered in genetics studies. Using in vitro and computational methods, we demonstrated that the Tyrp2 mutations strongly impacted protein folding and stability. Taking together our analyzed Tyr, Tyrp1, and Tyrp2 proteins and their OCA-related missense mutations using in vitro, in vivo, and in silico studies, we can be assisted in understanding the mechanisms of pathological mutations, which can lead to novel therapies for OCA1, OCA3, and OCA8 patients.

## 4. Materials and Methods

### 4.1. Protein’s Expression and Purification

Human Tyrp2 polypeptide contains 517 amino acids with a molecular weight of 58.51 kDa. Structural models of three mutant variants with deletions of the human Tyrp2 of 43-, 58-, and 63-residues at the C-terminus (labeled as Tyrp2_43tr_, Tyrp2_58tr_, and Tyrp2_63tr_, respectively) were obtained using molecular modeling, as shown in Figure S1. Three corresponding DNA constructs were generated to exclude the C-terminal transmembrane helix and prevent potential protein insolubility. Two missense variants, C40S and C61W, were created on the basis of the Tyrp2_43tr_ construct (residues 1-474) that demonstrated the best level of protein expression. All proteins were expressed in Baculovirus and commercially produced in whole insect *T.ni* larvae (Allotropic Tech, LLC, Halethorpe, MD, USA; https://allotropictech.com/; accessed on 20 January 2022), then purified by IMAC (His-Trap Crude 5 mL column) and GF (HiPrep 26/60 Sephacryl S-300, and Superdex 200 Increase GL 10/300 columns) chromatography using ÄKTAxpress and ÄKTA pure chromatography systems with the UNICORN software (GE Healthcare, Pittsburg, PA, USA) [14,18]. The GF columns were calibrated with the standards: thyroglobulin (670 kDa), γ-globulin (158 kDa), ovalbumin (44 kDa), myoglobin (17 kDa), and vitamin B12 (1.3 kDa) (Bio-Rad, Hercules, CA, USA). The protein concentration was determined using *A*260/280 nm measured with a NanoPhotometer N60-Touch (Implen, Westlake Village, CA, USA). The protein identity was confirmed by Western blot analysis using anti-Tyrp2 antibodies (B7, Santa Cruz Biotechnology, Dallas, TX, USA).

### 4.2. Dynamic Light Scattering

DLS experiments were performed using the Litesizer 500 (Anton Paar USA, Ashburn, VA, USA). Tyrp2 at 1 mg/mL in GF buffer (50 mM Tris-HCl (pH 7.4), 1 mM EDTA, 150 mM NaCl, 50 µM TCEP) was measured in a quartz cuvette QS 3.00 mm at 25 °C. Each run (measurement time) was 10 s, with continuous measurements, as the threshold number of counts was accumulated (10 × 10^6^). The intensity curve fittings and the hydrodynamic diameter were performed and calculated using the Kalliope 2.2.3 software (Anton Paar Kalliope Professional, Ashburn, VA, USA).

### 4.3. Copper and Zinc Content Analysis

Metal content analysis was performed commercially using a standard protocol for ICP-MS by Element Services Inc. (Santa Fe Springs, CA, USA). A total of 200 µL of samples (including Laboratory Fortified Blank, as quality control) were mixed with internal standards and then diluted to a mass of 2 g with a solution of 0.1% ammonium hydroxide, 0.05% EDTA, and 0.05% Triton X-10.

### 4.4. Tyrp2 Enzymatic Assay

Tyrp2 (1 mg/mL) was incubated with 1.5 mM L-DOPA (Sigma-Aldrich, Saint Louis, MO, USA) or dopachrome in a shaker at 270 rpm at 37 °C for 60 min. The absorption spectrum for the wavelength in the range of 200–900 was measured every 5 min using the Nano Photometer N60-Touch (Implen, Munich, Germany). Dopachrome was produced in the reaction of L-DOPA with Tyr immobilized to the magnetic beads, as previously described [21]. Briefly, magnetic beads (Advanced Biochemicals, Lawrenceville, GA, USA) were washed twice and resuspended in binding buffer (1 mM imidazole, 0.5 M NaCl, 20 mM Tris-HCl (pH 8.0)). Tyr (1 mg/mL) was then added to the suspension in a 1:1 ratio. The beads and protein were placed on an end-to-end rotator (Benchmark Scientific, Sayreville, NJ, USA) at RT for 30 min.

Following the binding incubation period, the supernatant was collected using a magnetic separator, and the beads were washed with wash buffer (5 mM imidazole, 0.25 M NaCl, 0.05% Tween-20, 10 mM Tris-HCl (pH 8.0)) and incubated at RT on an end-to-end rotator for 5 min. The beads were then resuspended in 1X PBS where 3 mM of L-DOPA (Sigma-Aldrich, Saint Louis, MO, USA) was added in a 1:1 ratio. This reaction was then placed in a shaker at 270 rpm at 37 °C for 10 min.

The isolated dopachrome was immediately used as a substrate for Tyrp2 to measure its enzymatic activity.

### 4.5. Intrinsic Tryptophan Fluorescence

Tryptophan fluorescence emission spectra of Tyrp2 (5 μM) were recorded in 10 mM sodium phosphate buffer (pH 7.4) in the presence or the absence of 100 mM TCEP or 8 M urea, using a SpectraMax i3 multimode detection platform (Molecular Devices, San Jose, CA, USA). Proteins were excited at 280 nm, and the emission was recorded from 300 to 400 nm. All fluorescence spectra were corrected for the buffer baseline.

### 4.6. Molecular Modeling

A tetrameric homology model of the intra-melanosomal domain (residues 14-480) of human Tyrp2 was built in YASARA (http://www.yasara.org/; accessed on 20 January 2022) using a single crystal structure of Tyrp1 as the structural template (PDB ID: 5M8L). Computer simulations were performed similar to that of published earlier [19,27]. The target sequence for human Tyrp2 was retrieved from UniProt (https://www.uniprot.org; accessed on 20 January 2022, ID: P40126). Each monomer was saved as a separate PDB file. YASARA did not build the active site zinc–water complex during modeling. Therefore, this was swapped into the monomer models from 5M8L. ZnA and ZnB atoms were bonded to the appropriate His residue NE2 atoms using the ‘CONECT’ record type in the PDB file of each model. The quality of each monomer was then evaluated using SAVES (https://www.doe-mbi.ucla.edu/services/; accessed on 20 January 2022). The highest scoring structure was refined with 500 ps of MD using YASARA’s ‘md_refine.mcr’ macro with the YASARA2 forcefield. PDB files were outputted every 25 ps during the simulation, and the final model was chosen from these using SAVES.

Two OCA8 mutations and one infantile nystagmus mutation (C40S, C61W, G59V, respectively) were modeled. Mutants were generated using the Edit > Swap > Residue function on the Tyrp2 homology model PDB file in YASARA. All three mutants and the original structure were subjected to 50 ns of MD using YASARA’s ‘run.mcr’ macro. Ion concentration was added as a mass fraction with 0.9% NaCl. The simulation temperature was set to 298 K with a water density of 0.997 g/m. The cell size extended to 10 Å beyond each side of the protein in the shape of a cube with dimensions 94.55 Å × 94.55 Å × 94.55 Å. Each simulation was run in YASARA using an AMBER14 forcefield, with a timestep of 2.5 fs. Simulation snapshots were outputted for every 0.1 ns, resulting in 500 sim-files for each simulation.

### 4.7. Global Computational Mutagenesis

Global computational mutagenesis was performed on the homology model of human recombinant Tyrp2, described in the previous paragraph. For any possible mutant variant of Tyrp2, a thermodynamic change in Gibbs free energy (ΔΔG) was calculated [28,29,30]. The changes were calculated by the semi-empirical method (FoldX) and standardized on a 0–1 scale as the unfolding parameter, or the fraction of protein in the unfolded state [29,30]. A foldability parameter is a sum of severity-weighted unfolding propensities for the 19 different mutations generated at each specific residue. Residues with the highest foldability of 19 were considered critical for protein stability [31]. The full-atomic Tyrp2 homology model and the results of the unfolding mutation screen are in the process to be available from the ocular proteome website at the NEI Commons (https://neicommons.nei.nih.gov/#/proteomeData/; accessed on 20 January 2022).

### 4.8. Molecular Docking

Models of Tyrp2 and C40S, C61W, and G59V mutants were created as described in Section 4.6. A 50 ns timeframe was selected from the MD simulation for each protein model. The 2D structure of the dopachrome ligand was retrieved in SDF format by using the PubChem compound database (http://pubchem.ncbi.nlm.nih.gov/; accessed on 20 January 2022). After the conversion of the file into 3D PDB format, energy minimization and ligand optimization were performed using the AMBER parameters with UCSF Chimera (1.15.0, UCSF, San Francisco, CA, USA). In total, 100, 200, and 500 steps of steepest descent minimization and 10, 20, and 50 steps of conjugate gradient minimization were performed. The molecular docking simulations were performed using the VINA software implemented in YASARA (21.8.27, IMBM, University of Graz, Austria). Briefly, the receptors and ligand files were used to set a target and the macro file ‘dock_run.mcr’ was run. A total of 25 VINA docking runs of the ligand to the receptor were sorted by binding energy (kcal/mol) and dissociation constant (pM) and then clustered, all differing by at least 5.0 Å heavy atom RMSD after superposing on the receptor. Docking results were only considered if the oxygens from the aromatic ring of dopachrome docked below 4.0 Å of the binuclear zinc active site. The further docked complexes were visualized using UCSF Chimera software.

## Figures and Tables

**Table 1:** Tyrp2 parameters were determined from the gel-filtration chromatography, dynamic light scattering, and ICP-MS analysis.

GF Chromatography			DLS				Metal Analysis	
Total Protein (mg)		MW (kDa)	H_D_ (nm)	Monomer Peak Diameter (nm)	PDI (%)	Intercept g1²	Zn (µg/g)	Cu (µg/g)
Lysate	Superdex 75							
4703 ± 543	0.54 ± 0.12	56.24 ± 1.30	7.76 ± 0.18	9.23 ± 0.39	21.39 ± 0.90	0.59 ± 0.01	0.07 ND* 0.64**	0.014 0.23* 0.008**

The total protein was estimated by the Warburg–Christian method using absorbance at 260/280 nm. To estimate the molecular weight of Tyrp2 (kDa), we pre-calibrated the Superdex 200 Increase 10/300 column with the GF Bio-Rad standards: thyroglobulin (670 kDa), γ-globulin (158 kDa), ovalbumin (44 kDa), myoglobin (17 kDa), and vitamin B12 (1.3 kDa). The hydrodynamic diameter (H_D_), monomer peak diameter, polydispersity index (PDI), and the intercept g1^2^ were calculated from DLS using the Kalliope Professional 2.2.3 software (Anton Paar, Graz, Austria). Metal analysis was done using ICP-MS analysis. * Tyr; ** Tyrp1.

**Table 2:** Global mutagenesis of Tyrp2 homology model, recombinant Tyrp2 purification yield, and clinical significance of mutation from ClinVar (https://www.ncbi.nlm.nih.gov/clinvar; accessed on 20 January 2022).

Mutation OCA8	Unfolding Fraction	Foldability	ΔΔG kcal/mol	ClinVar	Protein Purification Yield
C40S	1	19	2.75	Pathogenic/likely pathogenic	~0
G59V	1	19	77.6	n/a	n/a
C61W	0.99	19	6.01	Pathogenic/likely pathogenic	~0

## Data Availability

Data available in Supplementary Materials.

## Supplementary Materials

The following supporting information can be downloaded athttps://www.mdpi.com/article/10.3390/ijms23031305/s1.

## Abbreviations

Tyr: tyrosinase; Tyrp1, tyrosinase-related protein 1; Tyrp2, tyrosinase-related protein 2; DCT, dopachrome tautomerase; OCA, oculocutaneous albinism; OCA8, oculocutaneous albinism type 8; C40S, G59V, and C61W, OCA8-related mutant variants; Cys-rich, cysteine-rich domain; EGF, EGF-like domain; IMAC, immobilized metal affinity chromatography; GF, gel-filtration chromatography; DLS, dynamic light scattering; DHICA, 5,6-dihydroxyindole-2-carboxylic acid; DHI, 5,6-dihydroxyindole; IQCA, indole-5,6-quinone-2-carboxylic acid; ROS, reactive oxygen species; TCEP, tris(2-carboxyethyl)phosphine; DTT, 1,4-dithiothreitol; β-ME, β-mercaptoethanol.
