# Supplementary material for: Protein Biochemistry and Molecular Modeling of the Intra-Melanosomal Domain of Human Recombinant Tyrp2 Protein and OCA8-Related Mutant Variants
Source: Int J Mol Sci. 2022 Jan 24;23(3):1305. doi: 10.3390/ijms23031305 (PMC8836267; doi:10.3390/ijms23031305)
Supplement: Supplementary file 1 [file ijms-23-01305-s001.zip › ijms-1522598-supplementary.pdf]

Monika B. Dolinska, Taariq Woods, Isabella Osuna, and Yuri V. Sergeev.

Protein biochemistry and computational analysis of the intra-melanosomal domain of human recombinant Tyrp2 protein and OCA8-related mutant variants

Supplementary Material

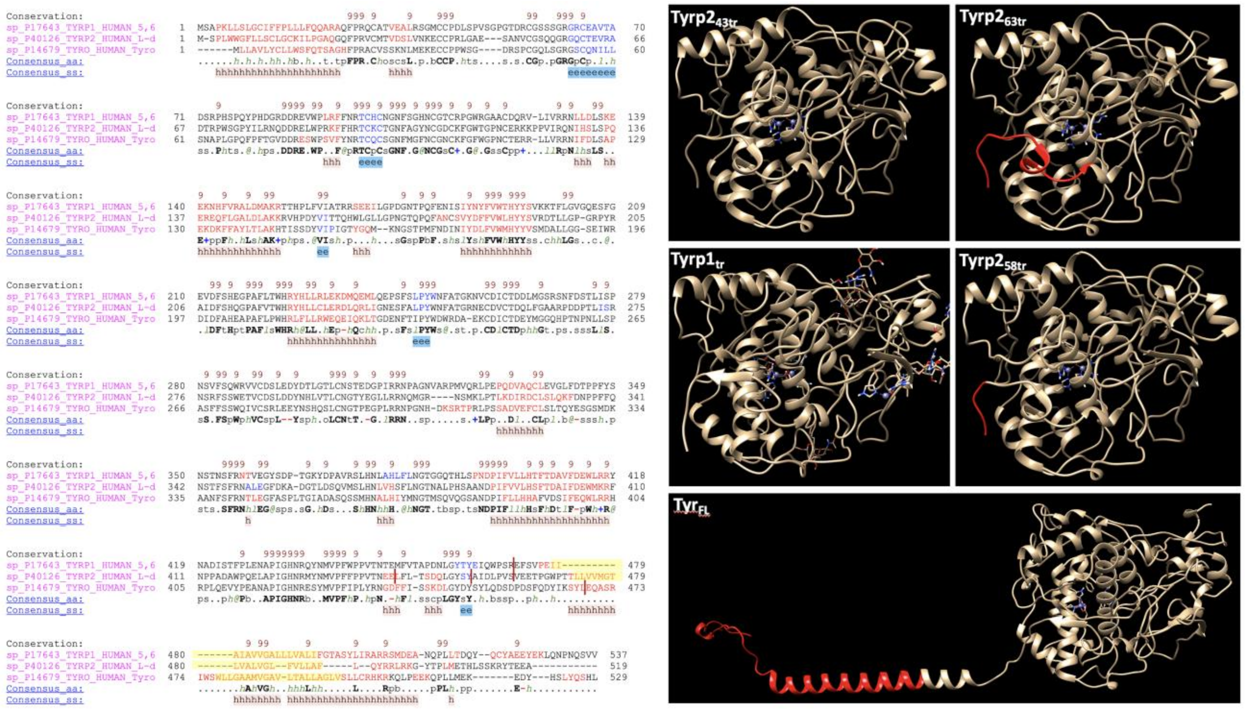

Supplementary Figure S1. The multiple-sequence alignment of human Tyr, Tyrp1, and Tyrp2 (left) and homology models of truncated recombinant variants of Tyrp2 and full-length recombinant tyrosinase (right).

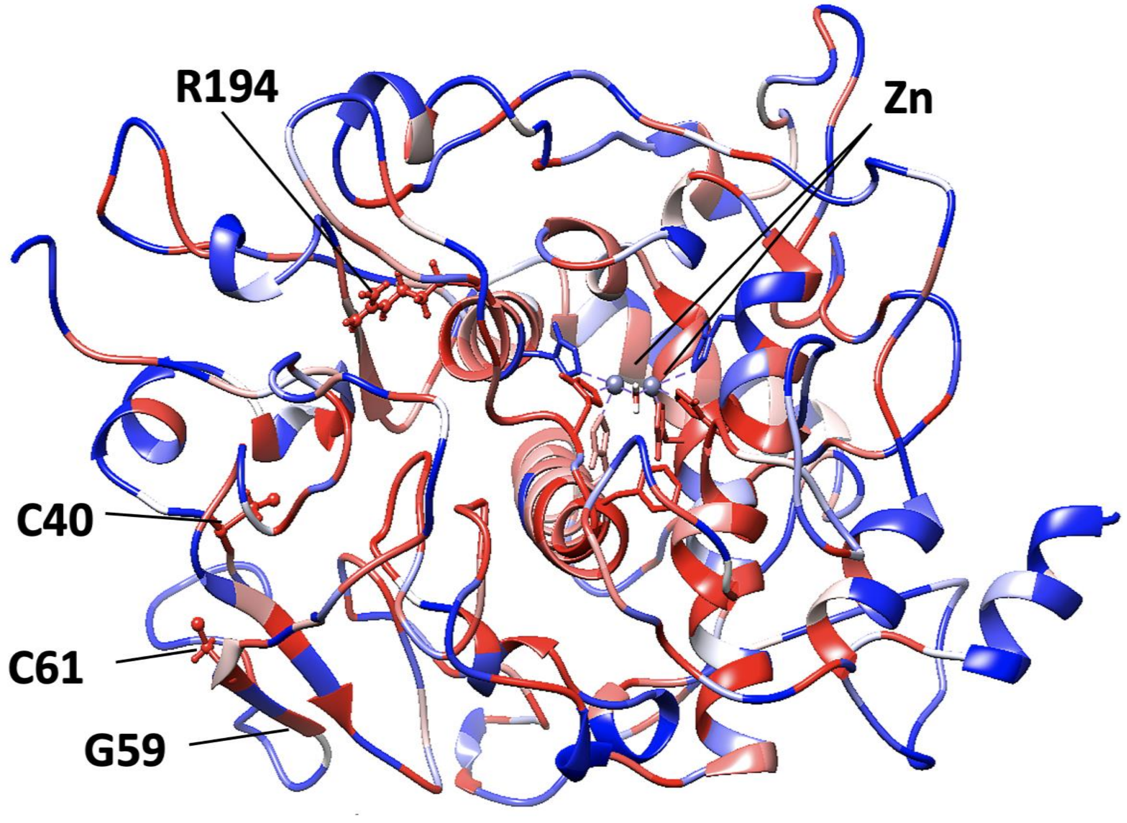

Supplementary Figure S2. Global computational mutagenesis of Tyrp2 homology model. Residues with the highest foldability when mutated are critical for protein stability (red). Residues of low foldability do not affect protein stability (blue). The positions 40, 59, 61, and 194 are affected by mutant variants and R194 (*slaty* mouse).

A

WT

	L166	L167	G168	P169	N170	G171
L44	1.3008	1.2507	1.7944	1.195	1.5499	0.8991
G45	1.6264	1.4628	1.8042	1.2173	1.6024	1.1376
A46	1.1913	1.3797	1.9597	1.4178	1.8181	0.9465
E47	0.5925	1.3226	2.0225	1.6398	1.8851	1.108
S48	0.8095	1.4037	2.2061	1.7796	2.0961	1.3636
A49	0.8829	1.3719	2.2545	1.8067	2.153	1.4673
N50	0.7067	1.0392	2.0057	1.509	1.8672	1.3124
V51	0.7006	1.1745	2.218	1.6793	2.0017	1.5326
C52	0.6646	1.1158	2.0591	1.568	1.9588	1.3224
G53	0.9556	1.5617	2.5034	1.9812	2.3475	1.6573
S54	0.6843	1.2737	2.3527	1.7763	2.1317	1.6383
Q55	0.6851	1.256	2.3741	1.8849	2.2369	1.8025
Q56	0.5664	0.8351	1.9131	1.354	1.7477	1.3018
G57	0.4313	0.6094	1.6185	1.1371	1.5346	1.1505
R58	0.5161	0.7773	1.6695	1.2472	1.6813	1.1066
G59	0.6159	0.7647	1.4844	1.1158	1.5888	0.8275
Q60	0.6423	0.7761	1.3757	1.0676	1.5968	0.7581
C61	0.8134	1.0046	1.4952	1.2646	1.8249	0.7049
T62	1.1153	1.3129	1.7444	1.512	1.9963	0.8023
E63	1.4991	1.7448	1.9629	1.776	2.1659	0.7833

C40S

	L166	L167	G168	P169	N170	G171
L44	2.2	2.6194	3.4484	2.8056	3.8205	3.0829
G45	3.8965	4.3793	5.1618	4.4846	5.5417	4.7846
A46	2.8859	3.4169	4.3369	3.7489	4.9513	3.8563
E47	3.0311	3.6489	4.6095	4.1299	5.3154	3.8583
S48	2.0847	2.5604	3.4715	3.0428	4.3692	3.0211
A49	2.0123	2.3948	3.2887	2.9475	4.2809	2.9215
N50	1.9797	2.2471	3.1237	2.8834	4.1714	2.8614
V51	1.7782	2.0777	3.0029	2.729	3.9725	2.7142
C52	2.0226	2.3782	3.3084	3.0514	4.362	2.9365
G53	1.946	2.3468	3.2784	2.9231	4.2068	2.8886
S54	1.9853	2.2986	3.2275	2.9273	4.1968	2.9325
Q55	1.9081	2.1509	3.0864	2.9019	4.1806	2.8246
Q56	1.7992	1.9929	2.9035	2.7311	3.9881	2.7102
G57	1.8734	2.0766	2.9345	2.7979	4.1121	2.7231
R58	2.0763	2.3366	3.2035	3.0678	4.4443	2.9112
G59	2.16	2.4349	3.2762	3.1555	4.5674	2.9694
Q60	1.9803	2.2143	3.012	3.0055	4.4258	2.7051
C61	1.8935	2.0805	2.8171	2.9189	4.3071	2.4887
T62	1.6756	1.736	2.4385	2.6078	3.9473	2.2678
E63	1.5625	1.5289	2.1861	2.4326	3.7564	2.1152

R-R Dist., St. Dev.: 5 / 2.5 / 0

B

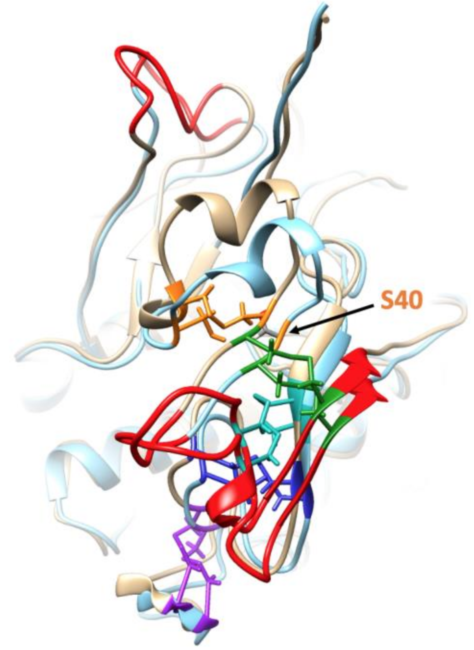

Supplementary Figure S3. Sections from residue-residue (RR) distance maps of Tyrp2 and C40S with visualized structure changes. **A**: Maps were generated for each structure across three-time points: 0, 25, and 50 ns. Displayed here are the standard deviations of distances between residues. The R27-P42 and L44-E63 regions within the EGF-like domain show increased fluctuations of distances between the neighboring G165-T172 loop in C40S compared to the wild-type. **B**: Tyrp2 (beige) and C40S (light blue) structures at 50 ns were superimposed with mapped regions highlighted in red. The introduction of Ser at position 40 prevents the formation of disulfide 1 (orange).

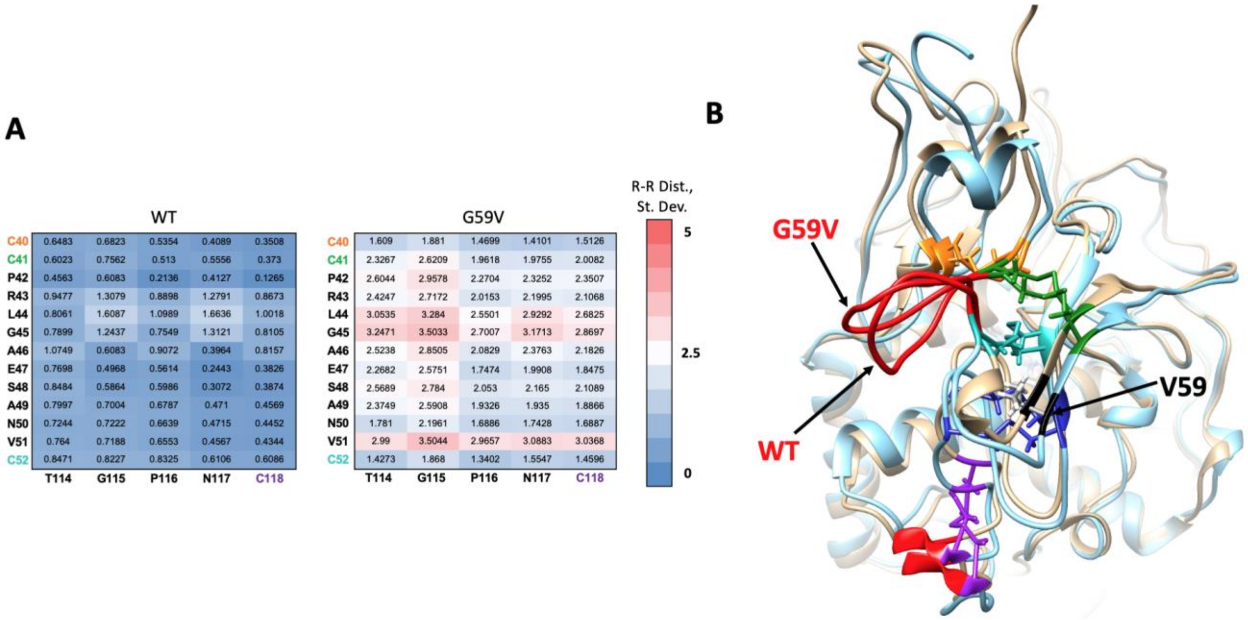

Supplementary Figure S4. RR distance maps and structure superposition of Tyrp2 and G59V. A: Maps were generated for each structure across three-time points: 0, 25, and 50 ns. Fluctuations appear most frequently but least severely in G59V compared to C40S and C61W, having the highest average standard deviation across the entire structure (0.947 Å) compared to the other mutants. Distances between EGF-like domain regions C40-C52 and T114-C118, which includes four disulfide-forming Cys residues, experience increased fluctuations in G59V. **B**: Superposition of Tyrp2 (beige) and G59V (light blue) at 50 ns showed shifts in mapped regions (red) and in the beta-sheet in which the G59V mutation is present (black).

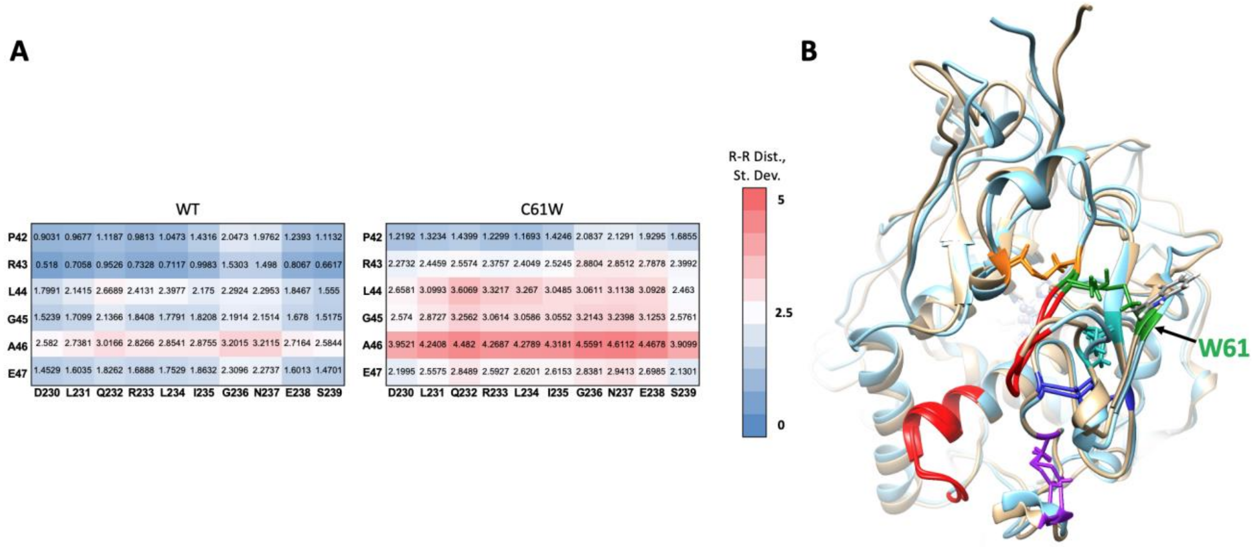

Supplementary Figure S5. R**R distance maps and structure superposition of Tyrp2 and C61W**. **A**: Maps were generated for each structure across three-time points: 0, 25, and 50 ns. Regions P42-E47 and D230-S239 experience increased degrees of distance fluctuations in C61W compared to the wild-type. **B**: Superposition of Tyrp2 (beige) and C61W (light blue) at 50 ns shows increased distance between mapped regions (red) and shortening of the tyrosinase domain helix in C61W. The alteration to Trp at position 61 prevents the formation of disulfide 2 (green).

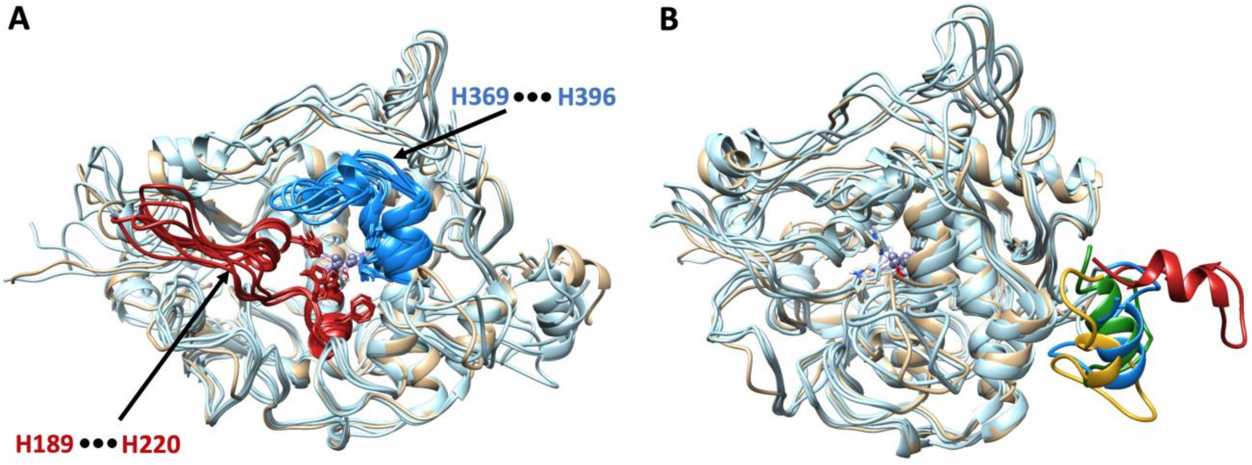

Supplementary Figure S6. Tyrp2 metal ion-binding domains and C-terminal shifts. **A**: Tyrp2 (beige) and all three mutant variants (light blue) at 50 ns were superimposed. Two domains make up the Tyrp2 active site, each containing three His residues essential for binding of ZnA and ZnB. ZnA binds His189, H211, and H220 of the first domain (red, H189●●●H220). ZnB binds His369, H373, and His396 of the second domain (blue, H369●●●H396). Movement in these regions was quantified and compared between Tyrp2 and mutant variants using RR distance maps. **B**: Tyrp2 (beige) and all three mutant variants (light blue) at 50 ns were superimposed. C-terminal residues (460-476) of Tyrp2, C40S, C61W, and G59V were colored red, yellow, blue, and green, respectively. This superposition displays prominent shifting of the C-terminal region that occurs during MD in Tyrp2 (**Supplementary Figure S7**, red area), but not in the mutants.

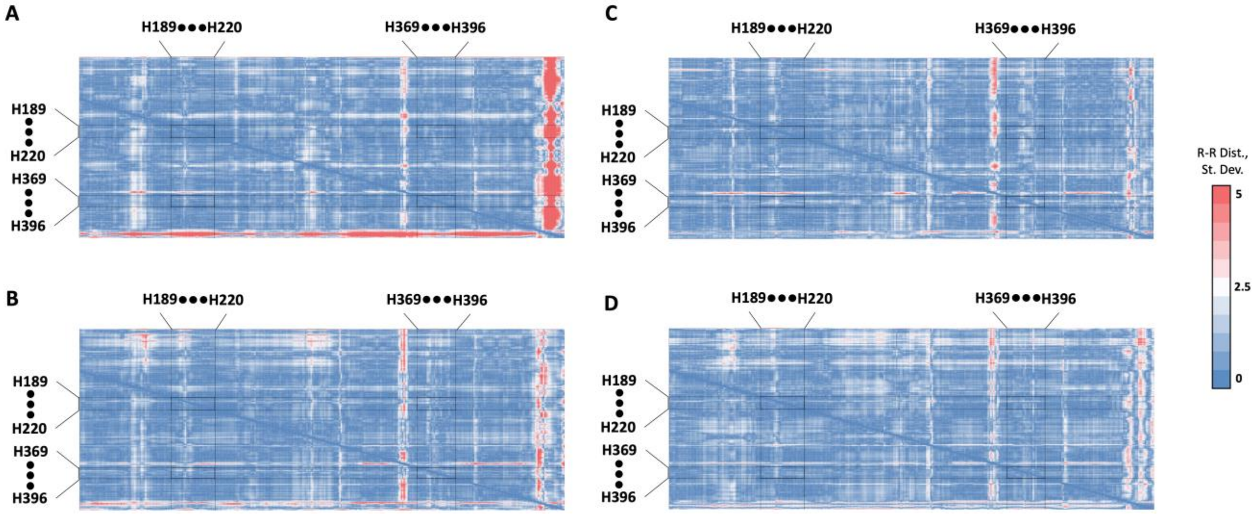

Supplementary Figure S7. Changes in metal ion-binding domains from residue-residue distance maps of Tyrp2 (A), C40S (B), C61W (C), and G59V (D). Maps were generated for each model at three timestamps: 0, 25, and 50 ns. These maps show standard deviations of residue distances. Map areas for domain 1 (H189●●●H220) and domain 2 (H369●●●H396) are outlined with black boxes (**Supplementary Figure 6A**). The four boxes where the areas intersect show the distance standard deviations between the two domains and between residues of the same domain. From these four boxes we see that there are no drastic changes in the binding domains between Tyrp2 and the mutant variants. The greatest increase in active site standard deviations occurred in C61W.

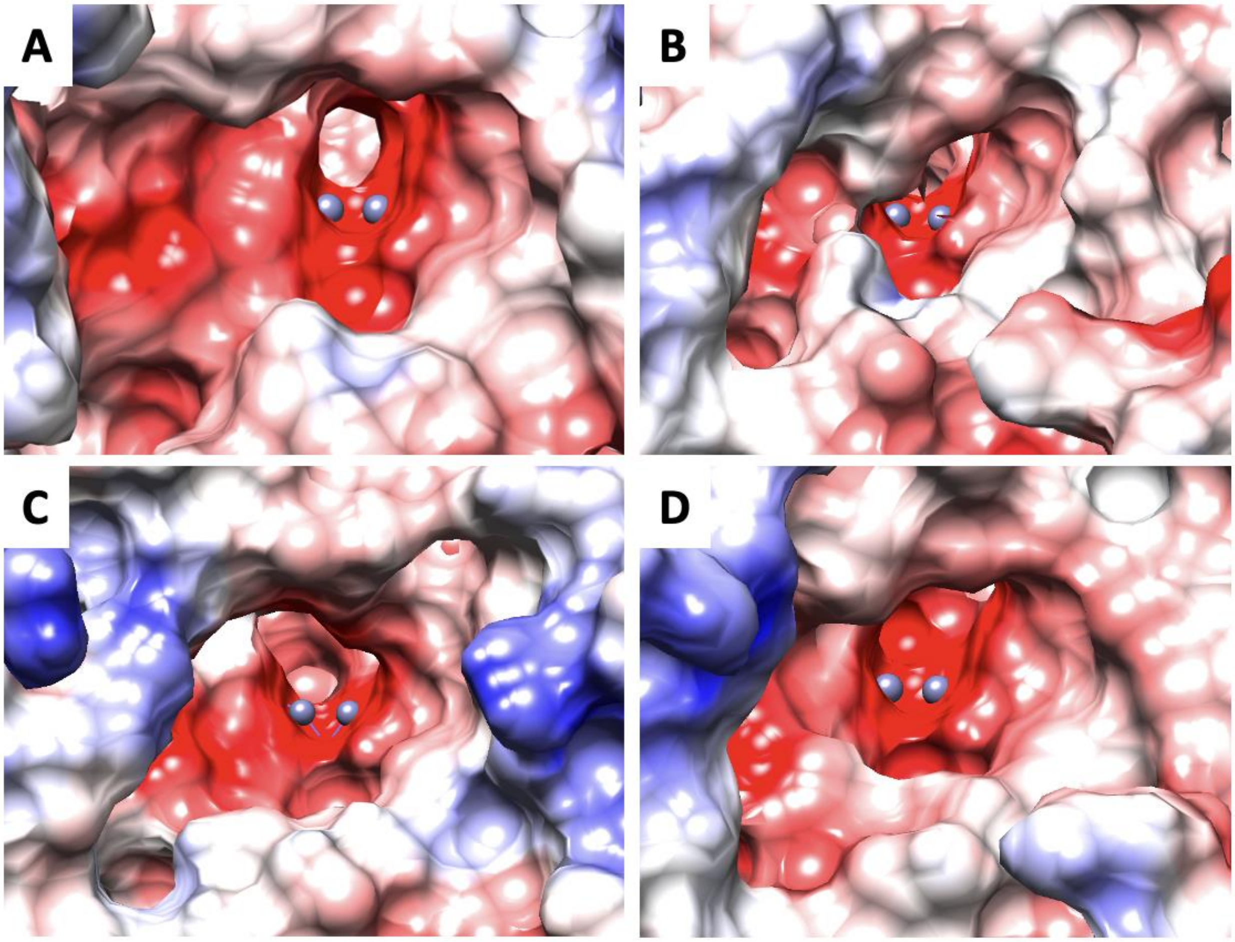

Supplementary Figure S8. The electrostatic potential of the molecular surfaces around the active sites of Tyrp2 (Panel A), C40S (Panel B), C61W (Panel C), and G59V (Panel D) based on the charges of the residues they enclose using the Coulombic Surface Coloring (red, -10, white, 0, blue, +10 kcal/mol*e).

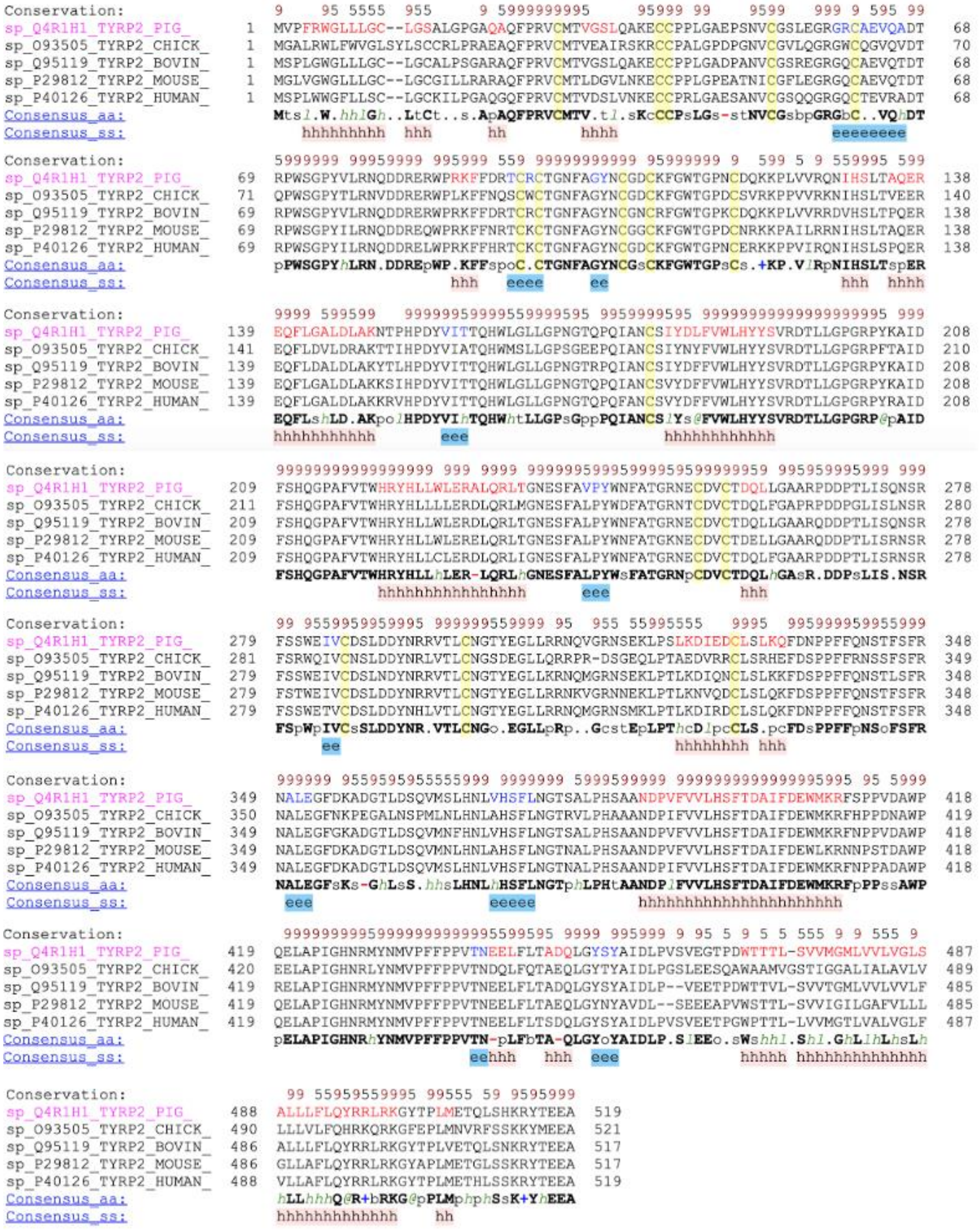

Supplementary Figure S9. The Tyrp2 cysteine residues are conserved in different species. The multiple amino acid sequence alignment of human, mouse, bovine, chick and pig Tyrp2 is shown. The alignment was performed using the PROMALS3D website (PROMALS3D - PROfile Multiple Alignment with Local Structure and 3D structure (swmed.edu)). Cysteine residues are highlighted by a yellow background.

Supplementary Table S1. Docked dopachrome interactions with Tyrp2.

Dopachrome pose	Binding energy (kcal/mol)	Dissociation constant Kd (µM)	Distance from zinc atoms (Å)		Contacting receptor residues	Ligand –receptor interactions		
			ZnA	ZnB		Hydrogen bonds	Hydrophobic	π - π
1	7.05	6.84	2.45	2.39	H189, H211, H369, N370, H373, S374, N380, A381, L382, A386, F392, H396	N370 - double	H373	H373
2	6.36	21.71	2.42	2.39	H189, H211, H369, N370, H373, N380, A381, L382, P383, A386, F392	A381	H373	H373
3	6.37	21.42	2.40	2.37	H189, H211, H369, N370, H373, N380, A381, L382, P383, A386, F392, H396	A381	H373	H373
	6.59 ± 0.40	16.66 ± 8.50	2.42 ± 0.03	2.38 ± 0.01	N/A	N/A	N/A	N/A

Supplementary Table S2. Docked dopachrome interactions with C61W.

Dopachrome pose	Binding energy (kcal/mol)	Dissociation constant Kd (µM)	Distance from zinc atoms (Å)		Contacting receptor residues	Ligand –receptor interactions		
			ZnA	ZnB		Hydrogen bonds	Hydrophobic	π - π
1	6.29	24.68	2.20	2.66	H189, H211, H369, N370, H373, S374, N380, A381, A386	-	H373	H369
2	5.90	47.50	2.45	2.21	H189, V193, D208, H211, H369, H373, N380, L382, P383, H384, A386, H396	-	H211	H211
3	6.32	23.46	2.20	2.66	H189, H211, H369, N370, H373, S374, N380, A381, L382, A386	-	H373	H369
	6.17 ± 0.23	31.88 ± 13.54	2.28 ± 0.14	2.51 ± 0.26	N/A	N/A	N/A	N/A

Supplementary Table S3. Docked dopachrome interactions with G59V.

Dopachrome pose	Binding energy (kcal/mol)	Dissociation constant Kd (uM)	Distance from zinc atoms (Å)		Contacting receptor residues	Ligand –receptor interactions		
			ZnA	ZnB		Hydrogen bonds	Hydrophobic	π - π
1	6.42	19.62	2.33	2.23	H189, H211, Q212, E352, F354, H369, N370, H373, N380, A381, P383, A386, H396	-	H211	H211
2	-	-	-	-	-	-	-	-
3	6.22	27.68	2.29	2.38	H189, H211, Q212, F354, H369, N370, H373, S374, A381, P383, A386, H396	N370	P383	H369
	6.32 ± 0.14	23.65 ± 5.70	2.31 ± 0.03	2.31 ± 0.11	N/A	N/A	N/A	N/A
